# 4-Chloro-2-hy­droxy-*N*-(4-methyl­phen­yl)benzamide

**DOI:** 10.1107/S1600536812000773

**Published:** 2012-01-14

**Authors:** Abdul Rauf Raza, Bushra Nisar, M. Nawaz Tahir, Sumaira Shamshad

**Affiliations:** a University of Sargodha, Department of Chemistry, Sargodha, Pakistan; b University of Sargodha, Department of Physics, Sargodha, Pakistan

## Abstract

In the title compound, C_14_H_12_ClNO_2_, the dihedral angle between the aromatic rings is 14.87 (11)° and an intra­molecular N—H⋯O hydrogen bond generates an *S*(6) ring. In the crystal, mol­ecules are linked by O—H⋯O hydrogen bonds, generating *C*(6) chains propagating along the *c*-axis direction.

## Related literature

For related structures, see: Raza *et al.* (2010 ▶, 2011 ▶). For graph-set notation, see: Bernstein *et al.* (1995 ▶).
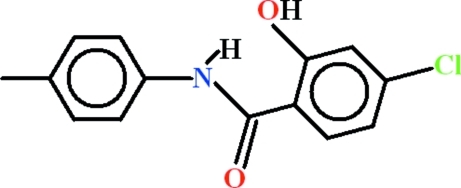


## Experimental

### 

#### Crystal data

C_14_H_12_ClNO_2_
*M* *_r_* = 261.70
Monoclinic, 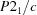

*a* = 13.8553 (12) Å
*b* = 7.6197 (7) Å
*c* = 12.0114 (11) Å
β = 104.937 (5)°
*V* = 1225.23 (19) Å^3^
*Z* = 4
Mo *K*α radiation
μ = 0.30 mm^−1^
*T* = 296 K
0.34 × 0.14 × 0.12 mm

#### Data collection

Bruker Kappa APEXII CCD diffractometer
Absorption correction: multi-scan (*SADABS*; Bruker, 2009) ▶ *T*_min_ = 0.979, *T*_max_ = 0.988
10366 measured reflections
2988 independent reflections
1832 reflections with *I* > 2σ(*I*)
*R*_int_ = 0.048

#### Refinement

*R*[*F* ^2^ > 2σ(*F* ^2^)] = 0.056
*wR*(*F* ^2^) = 0.163
*S* = 1.02
2988 reflections
165 parameters
H-atom parameters constrained
Δρ_max_ = 0.39 e Å^−3^
Δρ_min_ = −0.21 e Å^−3^

### 

Data collection: *APEX2* (Bruker, 2009 ▶); cell refinement: *SAINT* (Bruker, 2009 ▶); data reduction: *SAINT*; program(s) used to solve structure: *SHELXS97* (Sheldrick, 2008 ▶); program(s) used to refine structure: *SHELXL97* (Sheldrick, 2008 ▶); molecular graphics: *ORTEP-3 for Windows* (Farrugia, 1997 ▶) and *PLATON* (Spek, 2009 ▶); software used to prepare material for publication: *WinGX* (Farrugia, 1999 ▶) and *PLATON*.

## Supplementary Material

Crystal structure: contains datablock(s) global, I. DOI: 10.1107/S1600536812000773/hb6597sup1.cif

Structure factors: contains datablock(s) I. DOI: 10.1107/S1600536812000773/hb6597Isup2.hkl

Supplementary material file. DOI: 10.1107/S1600536812000773/hb6597Isup3.cml

Additional supplementary materials:  crystallographic information; 3D view; checkCIF report

## Figures and Tables

**Table 1:** Hydrogen-bond geometry (Å, °)

*D*—H⋯*A*	*D*—H	H⋯*A*	*D*⋯*A*	*D*—H⋯*A*
N1—H1⋯O1	0.86	1.96	2.658 (3)	138
O1—H1*A*⋯O2^i^	0.82	1.85	2.664 (2)	173

Symmetry code: (i) 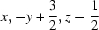.

**Fig. 1.**
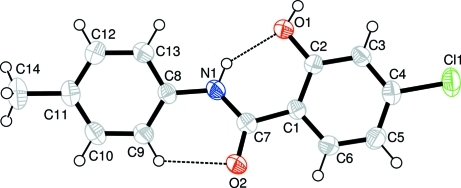
View of the title compound with displacement ellipsoids drawn at the 50% probability level. The dotted line indicate the intramolecular H-bond.

**Fig. 2.**
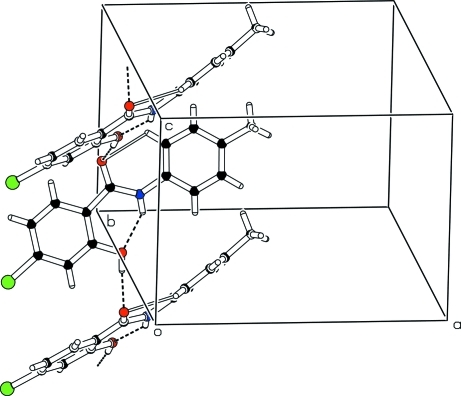
The partial packing showing polymeric chains extending along the c-axis.

## supplementary crystallographic information

### Comment

We have reported the crystal structures of (II) *i.e.*, 2-hydroxy-*N*-(4-methylphenyl)benzamide (Raza *et al.*, 2011) and (III) *i.e.*, *N*-(4-chlorophenyl)-2-hydroxybenzamide (Raza *et al.*, 2010) which are related to the title compound (I, Fig. 1). This compound has been prepared as a precursor for the synthesis of symmetric as well as asymmetric benzoxazepines.

In (I), the 3-chlorophenol group A (C1–C6/CL1/O1) and 4-methylanilinic group B (C8—C14) are roughly planar with r. m. s. deviations of 0.014 and 0.031 Å, respectively. The dihedral angle between A/B is 14.87 (11)°. The central formamide moiety C (O2/C7/N1) is of course planar. The dihedral angle between A/C and B/C is 7.59 (24)° and 18.74 (24)°, respectively. There exist intramolecular H-bondings of N—H···O and C—H···O types (Table 1, Fig. 1) completing S(6) ring motifs (Bernstein *et al.*, 1995). There exists inter-molecular H-bondings of O—H···O type (Table 1, Fig. 2) due to which the molecules are linked in the form of one dimensional polymeric chains extending along the crystallographic *c* axis.

### Experimental

The solution of 4-methylaniline (0.38 g, 4.0 mmol, 0.75 eq) in dry CHCl_3_ and dry Et_3_N (1 ml, 0.73 g, 7.0 mmol, 1.5 eq) was added slowly at room temperature to a mixture of 4-chloro-2-hydroxybenzoic acid (0.83 g, 5.0 mmol, 1 eq), SOCl_2_ (3.24 ml, 5.28 g, 44.0 mmol, 1.2 eq) and catalytic amount of dimethylformamide (1 drop) followed by 4 h reflux. After completion of reaction, the reaction mixture was cooled to room temperature, neutralized with aqueous NaHCO_3_ (10%), extracted with CHCl_3_ (3×25 ml). The organic layer was combined, dried over anhydrous Na_2_SO_4_, filtered and concentrated under reduced pressure to afford crude product. The column chromatographic purification with 5% EtOAc in hexane (2 *L*) over a silica gel packed column (23 cm length) afforded the title compound I as a white crystalline solid in the 18–59^th^ fractions (50 ml each).

### Refinement

Although H atoms were appeared in difference Fourier map but were positioned geometrically with (O–H = 0.82, N–H = 0.86 and C–H = 0.93–0.96 Å) and refined as riding with *U*_iso_(H) = *xU*_eq_(C), where *x* = 1.5 for hydroxy & methyl H-atoms and *x* = 1.2 for other H atoms.

### Crystal data

**Table:**

C_14_H_12_ClNO_2_	*F*(000) = 544
*M**_r_* = 261.70	*D*_x_ = 1.419 Mg m^−^^3^
Monoclinic, *P*2_1_/*c*	Mo *K*α radiation, λ = 0.71073 Å
Hall symbol: -P 2ybc	Cell parameters from 1243 reflections
*a* = 13.8553 (12) Å	θ = 1.1–27.9°
*b* = 7.6197 (7) Å	µ = 0.30 mm^−^^1^
*c* = 12.0114 (11) Å	*T* = 296 K
β = 104.937 (5)°	Block, colorless
*V* = 1225.23 (19) Å^3^	0.34 × 0.14 × 0.12 mm
*Z* = 4	

### Data collection

**Table:**

Bruker Kappa APEXII CCD diffractometer	2988 independent reflections
Radiation source: fine-focus sealed tube	1832 reflections with *I* > 2σ(*I*)
graphite	*R*_int_ = 0.048
Detector resolution: 7.6 pixels mm^-1^	θ_max_ = 28.3°, θ_min_ = 3.0°
ω scans	*h* = −18→17
Absorption correction: multi-scan (*SADABS*; Bruker, 2009)	*k* = −10→10
*T*_min_ = 0.979, *T*_max_ = 0.988	*l* = −15→15
10366 measured reflections	

### Refinement

**Table:**

Refinement on *F*^2^	Primary atom site location: structure-invariant direct methods
Least-squares matrix: full	Secondary atom site location: difference Fourier map
*R*[*F*^2^ > 2σ(*F*^2^)] = 0.056	Hydrogen site location: inferred from neighbouring sites
*wR*(*F*^2^) = 0.163	H-atom parameters constrained
*S* = 1.02	*w* = 1/[σ^2^(*F*_o_^2^) + (0.0803*P*)^2^ + 0.1504*P*] where *P* = (*F*_o_^2^ + 2*F*_c_^2^)/3
2988 reflections	(Δ/σ)_max_ < 0.001
165 parameters	Δρ_max_ = 0.39 e Å^−^^3^
0 restraints	Δρ_min_ = −0.21 e Å^−^^3^

### Special details

**Table:**

Geometry. Bond distances, angles *etc*. have been calculated using the rounded fractional coordinates. All su's are estimated from the variances of the (full) variance-covariance matrix. The cell e.s.d.'s are taken into account in the estimation of distances, angles and torsion angles
Refinement. Refinement of *F*^2^ against ALL reflections. The weighted *R*-factor *wR* and goodness of fit *S* are based on *F*^2^, conventional *R*-factors *R* are based on *F*, with *F* set to zero for negative *F*^2^. The threshold expression of *F*^2^ > σ(*F*^2^) is used only for calculating *R*-factors(gt) *etc*. and is not relevant to the choice of reflections for refinement. *R*-factors based on *F*^2^ are statistically about twice as large as those based on *F*, and *R*- factors based on ALL data will be even larger.

### Fractional atomic coordinates and isotropic or equivalent isotropic displacement parameters (Å^2^)

**Table:**

	*x*	*y*	*z*	*U*_iso_*/*U*_eq_	
Cl1	−0.34725 (5)	0.76527 (10)	−0.20816 (6)	0.0577 (3)	
O1	0.02457 (12)	0.6924 (3)	−0.03254 (14)	0.0433 (6)	
O2	0.00477 (12)	0.9612 (3)	0.26357 (13)	0.0429 (6)	
N1	0.11035 (14)	0.8338 (3)	0.17119 (16)	0.0362 (7)	
C1	−0.06586 (16)	0.8587 (3)	0.07420 (18)	0.0307 (7)	
C2	−0.06343 (17)	0.7590 (3)	−0.02354 (19)	0.0309 (7)	
C3	−0.15057 (17)	0.7321 (3)	−0.1100 (2)	0.0369 (8)	
C4	−0.23926 (18)	0.8024 (3)	−0.1004 (2)	0.0395 (8)	
C5	−0.24347 (18)	0.9064 (4)	−0.0066 (2)	0.0433 (9)	
C6	−0.15738 (17)	0.9312 (3)	0.0788 (2)	0.0378 (8)	
C7	0.01973 (17)	0.8882 (3)	0.17747 (19)	0.0330 (7)	
C8	0.20016 (17)	0.8321 (3)	0.25990 (19)	0.0336 (7)	
C9	0.21756 (18)	0.9335 (3)	0.3585 (2)	0.0398 (8)	
C10	0.30691 (19)	0.9134 (4)	0.4426 (2)	0.0442 (9)	
C11	0.38017 (19)	0.7971 (4)	0.4309 (2)	0.0447 (9)	
C12	0.36287 (19)	0.7055 (4)	0.3288 (2)	0.0498 (9)	
C13	0.27424 (19)	0.7211 (3)	0.2442 (2)	0.0440 (8)	
C14	0.4741 (2)	0.7698 (4)	0.5263 (3)	0.0643 (11)	
H1	0.11427	0.79538	0.10518	0.0435*	
H1A	0.01808	0.65337	−0.09769	0.0649*	
H3	−0.14878	0.66625	−0.17466	0.0443*	
H5	−0.30317	0.95784	−0.00191	0.0519*	
H6	−0.16000	0.99904	0.14229	0.0454*	
H9	0.17007	1.01407	0.36852	0.0478*	
H10	0.31781	0.98082	0.50924	0.0531*	
H12	0.41224	0.63092	0.31648	0.0598*	
H13	0.26452	0.65658	0.17647	0.0528*	
H14A	0.52971	0.75280	0.49345	0.0964*	
H14B	0.46641	0.66814	0.57041	0.0964*	
H14C	0.48596	0.87099	0.57550	0.0964*	

### Atomic displacement parameters (Å^2^)

**Table:**

	*U*^11^	*U*^22^	*U*^33^	*U*^12^	*U*^13^	*U*^23^
Cl1	0.0388 (4)	0.0747 (6)	0.0499 (4)	−0.0043 (3)	−0.0061 (3)	−0.0010 (4)
O1	0.0351 (9)	0.0606 (12)	0.0338 (10)	0.0033 (8)	0.0084 (7)	−0.0120 (9)
O2	0.0414 (10)	0.0602 (12)	0.0272 (9)	0.0023 (9)	0.0092 (7)	−0.0060 (8)
N1	0.0368 (11)	0.0452 (13)	0.0260 (10)	−0.0010 (9)	0.0068 (8)	−0.0022 (9)
C1	0.0343 (12)	0.0321 (13)	0.0252 (12)	−0.0021 (10)	0.0070 (9)	0.0041 (10)
C2	0.0314 (12)	0.0324 (13)	0.0294 (12)	−0.0004 (9)	0.0089 (9)	0.0042 (10)
C3	0.0388 (13)	0.0418 (15)	0.0294 (13)	−0.0043 (11)	0.0078 (10)	−0.0026 (10)
C4	0.0348 (13)	0.0446 (15)	0.0358 (14)	−0.0041 (11)	0.0034 (10)	0.0053 (11)
C5	0.0346 (14)	0.0503 (17)	0.0444 (15)	0.0045 (11)	0.0093 (11)	0.0018 (12)
C6	0.0395 (13)	0.0400 (14)	0.0341 (13)	0.0025 (11)	0.0098 (10)	−0.0020 (11)
C7	0.0386 (13)	0.0331 (13)	0.0284 (12)	−0.0039 (10)	0.0108 (10)	0.0054 (10)
C8	0.0352 (12)	0.0374 (14)	0.0276 (12)	−0.0045 (10)	0.0071 (10)	0.0026 (10)
C9	0.0386 (14)	0.0428 (15)	0.0374 (14)	−0.0022 (11)	0.0088 (11)	−0.0047 (11)
C10	0.0453 (15)	0.0510 (17)	0.0338 (14)	−0.0094 (12)	0.0056 (11)	−0.0053 (12)
C11	0.0385 (14)	0.0516 (16)	0.0394 (15)	−0.0056 (12)	0.0017 (11)	0.0051 (12)
C12	0.0364 (14)	0.0592 (18)	0.0513 (17)	0.0051 (12)	0.0066 (12)	−0.0047 (14)
C13	0.0418 (14)	0.0518 (16)	0.0381 (14)	−0.0005 (12)	0.0098 (11)	−0.0089 (12)
C14	0.0489 (18)	0.074 (2)	0.057 (2)	−0.0018 (15)	−0.0098 (14)	0.0058 (16)

### Geometric parameters (Å, °)

**Table:**

Cl1—C4	1.731 (3)	C9—C10	1.390 (4)
O1—C2	1.351 (3)	C10—C11	1.382 (4)
O2—C7	1.238 (3)	C11—C12	1.377 (4)
O1—H1A	0.8200	C11—C14	1.510 (4)
N1—C7	1.343 (3)	C12—C13	1.383 (4)
N1—C8	1.414 (3)	C3—H3	0.9300
N1—H1	0.8600	C5—H5	0.9300
C1—C7	1.496 (3)	C6—H6	0.9300
C1—C6	1.397 (3)	C9—H9	0.9300
C1—C2	1.406 (3)	C10—H10	0.9300
C2—C3	1.390 (3)	C12—H12	0.9300
C3—C4	1.372 (3)	C13—H13	0.9300
C4—C5	1.391 (3)	C14—H14A	0.9600
C5—C6	1.371 (3)	C14—H14B	0.9600
C8—C13	1.380 (3)	C14—H14C	0.9600
C8—C9	1.382 (3)		
			
C2—O1—H1A	109.00	C10—C11—C12	116.9 (2)
C7—N1—C8	127.9 (2)	C12—C11—C14	121.6 (3)
C7—N1—H1	116.00	C11—C12—C13	121.9 (3)
C8—N1—H1	116.00	C8—C13—C12	120.2 (2)
C2—C1—C6	117.6 (2)	C2—C3—H3	120.00
C2—C1—C7	126.1 (2)	C4—C3—H3	120.00
C6—C1—C7	116.2 (2)	C4—C5—H5	121.00
C1—C2—C3	120.0 (2)	C6—C5—H5	121.00
O1—C2—C1	119.1 (2)	C1—C6—H6	119.00
O1—C2—C3	120.9 (2)	C5—C6—H6	119.00
C2—C3—C4	120.3 (2)	C8—C9—H9	120.00
Cl1—C4—C3	119.57 (18)	C10—C9—H9	120.00
Cl1—C4—C5	119.4 (2)	C9—C10—H10	119.00
C3—C4—C5	121.1 (2)	C11—C10—H10	119.00
C4—C5—C6	118.3 (2)	C11—C12—H12	119.00
C1—C6—C5	122.6 (2)	C13—C12—H12	119.00
N1—C7—C1	117.4 (2)	C8—C13—H13	120.00
O2—C7—C1	119.5 (2)	C12—C13—H13	120.00
O2—C7—N1	123.1 (2)	C11—C14—H14A	109.00
N1—C8—C9	124.5 (2)	C11—C14—H14B	109.00
N1—C8—C13	116.3 (2)	C11—C14—H14C	110.00
C9—C8—C13	119.2 (2)	H14A—C14—H14B	109.00
C8—C9—C10	119.2 (2)	H14A—C14—H14C	109.00
C9—C10—C11	122.5 (2)	H14B—C14—H14C	110.00
C10—C11—C14	121.5 (2)		
			
C8—N1—C7—O2	6.7 (4)	C2—C3—C4—Cl1	−179.23 (18)
C8—N1—C7—C1	−173.7 (2)	C2—C3—C4—C5	2.2 (4)
C7—N1—C8—C9	−20.6 (4)	Cl1—C4—C5—C6	178.8 (2)
C7—N1—C8—C13	159.8 (2)	C3—C4—C5—C6	−2.7 (4)
C6—C1—C2—O1	177.7 (2)	C4—C5—C6—C1	1.1 (4)
C6—C1—C2—C3	−1.5 (3)	N1—C8—C9—C10	176.5 (2)
C7—C1—C2—O1	−5.5 (4)	C13—C8—C9—C10	−3.9 (4)
C7—C1—C2—C3	175.4 (2)	N1—C8—C13—C12	−177.2 (2)
C2—C1—C6—C5	1.0 (4)	C9—C8—C13—C12	3.1 (4)
C7—C1—C6—C5	−176.2 (2)	C8—C9—C10—C11	0.9 (4)
C2—C1—C7—O2	−171.4 (2)	C9—C10—C11—C12	2.7 (4)
C2—C1—C7—N1	8.9 (3)	C9—C10—C11—C14	−176.4 (3)
C6—C1—C7—O2	5.5 (3)	C10—C11—C12—C13	−3.5 (4)
C6—C1—C7—N1	−174.1 (2)	C14—C11—C12—C13	175.6 (3)
O1—C2—C3—C4	−179.2 (2)	C11—C12—C13—C8	0.7 (4)
C1—C2—C3—C4	−0.1 (3)		

### Hydrogen-bond geometry (Å, °)

**Table:**

*D*—H···*A*	*D*—H	H···*A*	*D*···*A*	*D*—H···*A*
N1—H1···O1	0.86	1.96	2.658 (3)	138
O1—H1A···O2^i^	0.82	1.85	2.664 (2)	173

Symmetry codes: (i) *x*, −*y*+3/2, *z*−1/2.

## References

[bb1] Bernstein, J., Davis, R. E., Shimoni, L. & Chang, N.-L. (1995). *Angew. Chem. Int.* Ed. Engl. **34**, 1555–1573.

[bb2] Bruker (2009). *APEX2*, *SAINT* and *SADABS* Bruker AXS Inc. Madison, Wisconsin, USA.

[bb3] Farrugia, L. J. (1997). *J. Appl. Cryst.* **30**, 565.

[bb4] Farrugia, L. J. (1999). *J. Appl. Cryst.* **32**, 837–838.

[bb5] Raza, A. R., Nisar, B. & Tahir, M. N. (2011). *Acta Cryst.* E**67**, o2253. 10.1107/S1600536811030716 PMC3200594 22065065

[bb6] Raza, A. R., Nisar, B., Tahir, M. N. & Shamshad, S. (2010). *Acta Cryst.* E**66**, o2922. 10.1107/S1600536810042030 PMC3009058 21589096

[bb7] Sheldrick, G. M. (2008). *Acta Cryst.* A**64**, 112–122. 10.1107/S0108767307043930 18156677

[bb8] Spek, A. L. (2009). *Acta Cryst.* D**65**, 148–155. 10.1107/S090744490804362X PMC2631630 19171970

